# HMGA1 Plays a Role in Counteracting DNA Damage Induced by BoHV-1 Productive Infection

**DOI:** 10.3390/ijms252413265

**Published:** 2024-12-10

**Authors:** Heci Zhao, Xiaotian Fu, Xiuyan Ding, Liqian Zhu

**Affiliations:** College of Life Sciences, Hebei University, Baoding 071002, China; 17803088063@163.com (H.Z.); fxt950828@163.com (X.F.)

**Keywords:** bovine herpesvirus 1, DNA damage, 53BP1, HMGA1

## Abstract

Bovine herpesvirus 1 (BoHV-1) productive infection induces the generation of DNA double-strand breaks (DSBs), which may consequently lead to cell apoptosis. In response to DSBs, the DNA damage repair-related protein 53BP1 is recruited to the sites of DSBs, leading to the formation of 53BP1foci, which are crucial for the repair of damaged DNA and maintaining genomic integrity by repairing DSBs. In this study, we discovered that HMGA1 may play a significant role in counteracting virus infection-induced DNA damage, as the siRNA-mediated knockdown of HMGA1 protein expression or inhibition of HMGA1 activity by the chemical inhibitor Netropsin uniformly exacerbates the DNA damage induced by BoHV-1 productive infection. Interestingly, HMGA1 may positively regulate 53BP1 expression, and treatment with Netropsin reduced the accumulation of 53BP1 protein in the nucleus, suggesting that HMGA1 may potentially influence 53BP1’s nuclear localization. However, this effect was reversed in the context of virus infection. Furthermore, Netropsin treatment restored the disruption of 53BP1 foci caused by virus infection, which is consistent with our findings that Netropsin enhances the nuclear accumulation of 53BP1. Collectively, these results indicate that HMGA1 is involved in countering DNA damage induced by virus infection. HMGA1 does indeed modulate the nuclear accumulation of 53BP1 protein, but this effect is counteracted by virus infection. Therefore, the biological function of HMGA1 in countering virus infection-induced DNA damage may be independent of its regulation of 53BP1 signaling. This is the first report suggesting that HMGA1 may be implicated in virus infection-induced DNA damage, although the precise mechanism remains to be elucidated. Furthermore, we report for the first time an interaction between HMGA1 and 53BP1, which is disrupted following virus infection.

## 1. Introduction

Bovine herpesvirus 1 (BoHV-1), an enveloped DNA virus, is a member of the family Herpesviridae and the subfamily Alphaherpesvirinae [1]. This virus is an important pathogen in cattle, known for causing severe diseases, such as the bovine respiratory disease complex (BRDC) [2], and viral abortion [1,3]. The prevalence of BoHV-1 in cattle herds worldwide has a substantial economic impact on the cattle industry. In particular, it is estimated to cost the US cattle industry around 3 billion dollars annually [4].

High mobility group A1 (HMGA1) is a non-histone chromatin regulator that binds to the minor groove of AT-rich DNA sequences through three N-terminal basic domains known as “AT-hooks” [5]. It recruits multiple factors to establish transcriptional complexes, modulating gene expression by remodeling chromatin structure and regulating interactions between transcriptional regulatory proteins [6,7]. Accumulating studies suggest that the overexpression of HMGA1 is associated with the pathogenicity of various tumors [8], including colorectal cancer [9], gastric cancer [10,11], and breast cancer [12]. It has been proposed that overexpressed HMGA1 in human malignant neoplasias contributes to the progression of malignant transformation due to increased DNA damage [13]. However, another study reports that breast cancer cells overexpressing HMGA1 exhibit a faster recovery upon induction of DNA double-strand breaks (DSBs) [14], indicating a potentially controversial role for HMGA1 in DNA damage repair. We have previously reported that HMGA1 plays a significant role in BoHV-1 productive infection in cell cultures [15], although the mechanisms underlying this role in viral infection remain not fully understood. Given that BoHV-1 productive infection in cell cultures leads to overproduction of DSBs [16], which may consequently lead to cell death [17] and is detrimental to viral replication, we wondered whether HMGA1 plays a crucial role in countering the DNA damage induced by BoHV-1 infection.

53BP1 is a chromatin-binding protein that plays a critical role in repairing DSBs by suppressing nucleolytic resection of DNA termini [18]. We have recently revealed that BoHV-1 productive infection alters the nuclear localization of 53BP1 both in vitro and in vivo, and disrupts the formation of 53BP1 foci during the virus’ productive infection in MDBK cells (submitted to Veterinary Microbiology, under review). Consequently, the impairment of 53BP1-dependent DNA damage repair may partially account for the induction of DNA damage.

In this study, we discovered that HMGA1 plays a crucial role in mitigating DNA damage induced by BoHV-1 infection. Both the knockdown of HMGA1 protein expression and the inhibition of HMGA1 activity using the chemical inhibitor Netropsin uniformly exacerbated the DNA damage caused by BoHV-1 productive infection. Furthermore, our findings suggest that HMGA1 may be involved in the regulation of 53BP1 nuclear accumulation, which plays an important role in DNA damage repair. However, the biological function of HMGA1 in countering virus infection-induced DNA damage appears to be independent of its regulation of the 53BP1-dependent DNA damage repair. This is the first report suggesting that HMGA1 may be implicated in BoHV-1 infection-induced DNA damage, although the precise mechanism remains to be elucidated. Furthermore, we report for the first time an interaction between HMGA1 and 53BP1.

## 2. Results

### 2.1. BoHV-1 Productive Infection in MDBK Cells Leads to Increased Nuclear Accumulation of HMGA1 Protein

**Figure 1:**
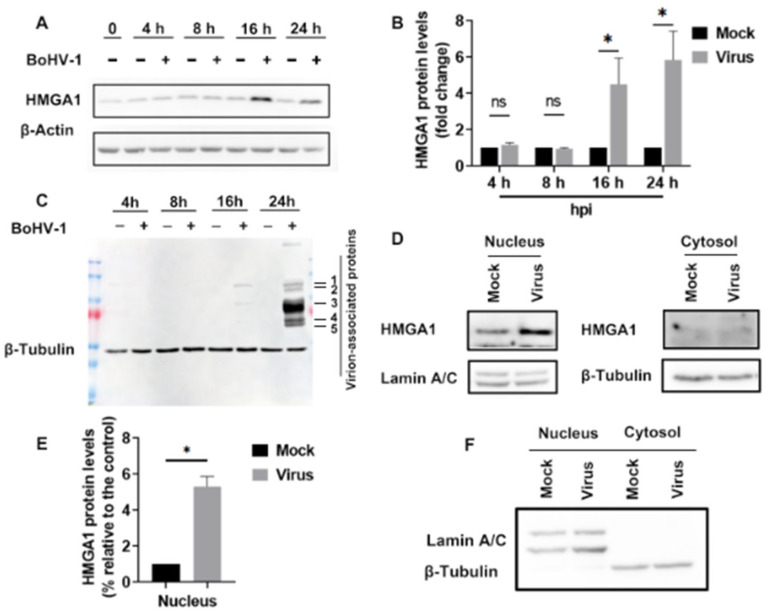
Enhanced accumulation of HMGA1 protein in the nucleus upon BoHV-1 productive infection in MDBK cells. (**A**,**C**) Confluent MDBK cells in 60 mm dishes were either mock-infected or infected with BoHV-1 at an MOI of 1. Infections were allowed to proceed for 4, 8, 16, and 24 h. Cell lysates were prepared to analyze protein expression of HMGA1 (**A**), and virion-associated proteins (**C**), using Western blot. Either β-Actin or β-Tubulin was used as a protein load control for subsequent quantitative analysis, respectively. (**B**,**E**) The band intensity of HMGA1 was quantitatively analyzed using the free software Image J (https://imagej.net/ij/download.html, accessed on 1 December 2020). The fold change in HMGA1 protein levels after infection was calculated relative to the mock-infected controls, which were set to a value of 1. Significance was assessed with a student’s *t*-test (* *p* < 0.05, ns, not significant). (**D**) Nuclear and cytosolic fractions were extracted from MDBK cells that were mock-infected or infected with BoHV-1 (MOI = 1) for 24 h using a commercial kit (Beyotime Biotechnology, Shanghai, China, cat# P0027). HMGA1 protein levels in both respective fractions were detected by Western blotting. (**F**) Lamin A/C, a nuclear protein marker, and β-tubulin, a cytoplasmic protein marker, were detected in both fractions by Western blot to confirm the purity of the nuclear and cytosolic extracts. Data shown are representative of three independent experiments.

To evaluate the role of HMGA1 protein in BoHV-1-induced DNA damage, we initially monitored its expression in MDBK cells infected with BoHV-1 at a multiplicity of infection (MOI) of 1 over various time points. We observed that BoHV-1 productive infection in MDBK cells resulted in increased HMGA1 protein expression at 12 and 24 h post-infection (Figure 1A), which corroborates our previous findings [15]. HMGA1 protein levels were elevated approximately 4.52- and 5.84-fold compared to the mock-infected control at these time points (Figure 1B). The expressions of virion-associated proteins, labeled as 1, 2, 3, 4, and 5 respectively, were clearly detected at 16 and 24 h post infection (hpi) (Figure 1C), confirming that virus infection accounts for the alteration of HMGA1 protein expression. Consequently, a 24 h virus infection period was chosen to analyze the subcellular localization of HMGA1 protein in response to BoHV-1 infection.

Upon further examination of HMGA1 accumulation in the nucleus and cytosol using a commercial nuclear protein extraction kit, we found that HMGA1 expression in the nucleus increased approximately 5.32-fold compared to the mock-infected control (Figure 1D,E). This increase in nuclear HMGA1 accumulation was consistent with the elevated expression in the whole cell extracts observed following virus infection. HMGA1 was rarely detected in the cytosolic fractions (Figure 1D). Overall, these results suggest that BoHV-1 productive infection promotes the nuclear accumulation of HMGA1. The specific expression of Lamin A/C in the nucleus and β-tubulin in the cytosol, but not in their respective counterpart fractions (Figure 1F), confirmed the expected purity of the fractions, thereby validating our findings that virus productive infection increases the nuclear accumulation of HMGA1 protein.

### 2.2. Netropsin Exacerbated DNA Damage Induced by BoHV-1 Infection

**Figure 2:**
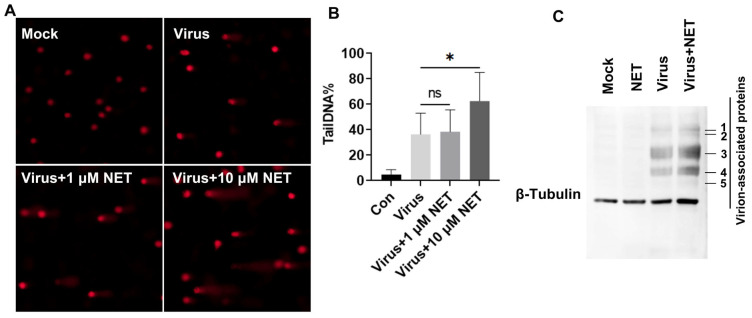
Impact of Netropsin on BoHV-1-induced DNA damage in MDBK cells. (**A**) MDBK cells were either mock-infected or infected with BoHV-1 at an MOI of 1 in the presence of either DMSO control or the HMGA1 inhibitor Netropsin at the indicated concentrations for 24 h. DNA damage was subsequently assessed using the comet assay, and images were captured under a fluorescence microscope. (**B**) A total of 300 cells were randomly selected from each sample for the analysis of TailDNA% using the CASP software (University of Wroclaw, Wrocław, Poland, http://www.casp.of.pl, accessed on 15 August 2015). Data are presented as means ± SD from three independent experiments. Statistical analyses were conducted using Student’s *t*-test (* *p* < 0.05; ns, not significant). (**C**) MDBK cells in 60 mm dishes were either mock-infected or infected with BoHV-1 at an MOI of 1 for 24 h in the presence of either DMSO control or Netropsin (10 μM). Then, cell lysates were prepared and subjected to detection of virion-associated proteins by Western blotting. Data shown are representative of three independent experiments.

There are no specific drugs capable of inhibiting HMGA1’s biological functions. Since HMGA1 specifically binds to the minor groove of the A/T-rich DNA motif, and Netropsin competitively binds to the minor groove of A/T-rich double-stranded DNA and interferes with the binding of HMGA1 and other high-mobility group AT-hook like proteins, such as HMGA2, to DNA [15]. We initially employed the inhibitor Netropsin to investigate the role of HMGA1 in DNA damage induced by BoHV-1 infection, utilizing comet assays, a standard method for visually detecting DSBs. As expected, BoHV-1 infection resulted in significant DNA damage, visible as prominent comet tails in infected MDBK cells (Figure 2A, upper right panel). This damage was exacerbated in cells treated with 10 μM Netropsin post-infection (Figure 2A, lower right panel). Quantifying DNA damage as the percentage of DNA fluorescence in the tail (tailDNA%) revealed that the tailDNA% was approximately 36.38% in virus-infected cells; it increased to approximately 62.55% in those exposed to Netropsin (Figure 2B). This indicates that Netropsin can exacerbate DNA damage induced by BoHV-1 infection. Notably, Netropsin, at a concentration of 50 μM, significantly reduces BoHV-1 productive infection, with the viral titer decreasing approximately 7-fold relative to the mock-treated control [15]. Here, at a concentration of 10 μM, Netropsin exhibits no significant effects on the expression of virion-associated proteins, as detected by Western blotting (Figure 2C). Thus, the increased DNA damage observed following Netropsin treatment is not attributable to changes in viral yields.

### 2.3. Knockdown of HMGA1 Protein Expression by siRNAs Exacerbated DNA Damage Induced by BoHV-1 Infection

**Figure 3:**
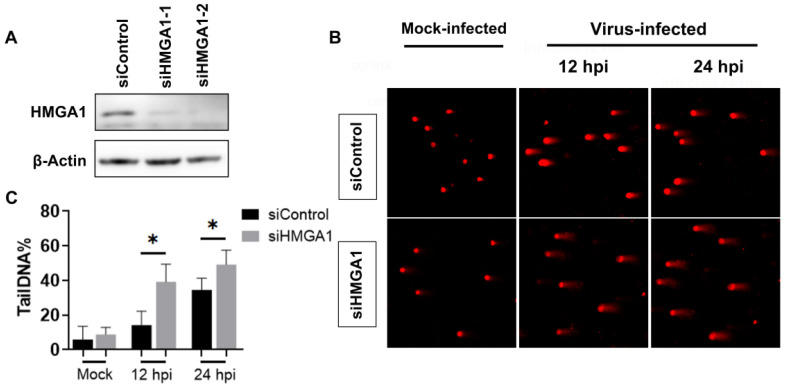
Impact of HMGA1 knockdown on BoHV-1-induced DNA damage in MDBK cells. (**A**) MDBK cells in 60 mm dishes were transfected with either scramble siRNA (siControl) or HMGA1-specific siRNAs (siHMGA1-1 and siHMGA1-2). After 48 h of transfection, cell lysates were prepared for the detection of HMGA1 by Western blot. β-Actin was used as a protein load control. Data shown are representative of three independent experiments. (**B**) MDBK cells were transfected with siControl or HMGA1-specific siRNAs (siHMGA1-1 and siHMGA1-2). Following 48 h of transfection, the cells were infected with BoHV-1 for 12 and 24 h. Then DNA damage was assessed using the comet assay, and images were captured under a fluorescence microscope. (**C**) Three hundred cells were randomly selected from each sample for the analysis of TailDNA% using the free software CASP. Data are presented as means ± SD from three independent experiments. Statistical analyses were conducted using Student’s *t*-test (* *p* < 0.05).

Considering that the chemical inhibitor Netropsin may have off-target effects, we employed siRNA-mediated knockdown of HMGA1 protein expression to investigate the role of HMGA1 in DNA damage induced by BoHV-1 infection. Two commercially available siRNAs, siRNAHMGA1-1 and siRNAHMGA1-2, significantly reduced HMGA1 protein levels compared to the siRNAControl (Figure 3A). We proceeded with siRNAHMGA1-1 for further studies. Comet assays conducted at 12 and 24 hpi showed more pronounced comet tails in virus-infected cells transfected with siRNAHMGA1 than in those transfected with siRNAControl (Figure 3B). The tailDNA% increased from approximately 14.26% and 34.54% in siRNAControl-transfected and virus-infected cells at 12 and 24 hpi, respectively, to 39.18% and 49.15% in siRNAHMGA1-1-transfected and virus-infected cells (Figure 3C). This indicates that, similar to the effects observed with Netropsin treatment, HMGA1 knockdown increases DNA damage or DSBs in virus-infected cells. We have previously reported that HMGA1-specific siRNAs reduced virus production approximately 3- to 5-fold relative to the scrambled siRNA [15]. So, the exacerbated DNA damage in MDBK cells with HMGA1 knockdown is not due to alterations in virus titers. In conclusion, these data imply that HMGA1 may play a significant role in mitigating DNA damage following BoHV-1 productive infection.

### 2.4. Netropsin Reversed the Disruption of 53BP1 Foci Induced by BoHV-1 Infection

**Figure 4:**
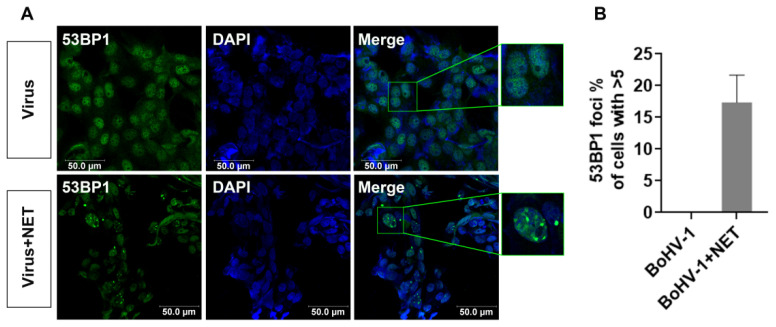
Restoration of 53BP1 foci formation by Netropsin in BoHV-1-infected MDBK cells. (**A**) MDBK cells were infected with BoHV-1 in the presence of either DMSO control or Netropsin (10 μM) for 24 h. Subsequently, an immunofluorescence assay (IFA) was performed to detect 53BP1 foci using a 53BP1-specific antibody. Nuclei were counterstained with DAPI. Images were captured using confocal microscopy. Zoomed-in images in green frames demonstrate the typical 53BP1 foci. (**B**) Approximately two hundred cells were selected to calculate the percentage of cells containing five or more foci per cell. Data are presented as means ± SD from three independent experiments.

When a DSB occurs, 53BP1, a chromatin-binding protein crucial for DSB repair by inhibiting nucleolytic resection of DNA termini, is rapidly recruited to the vicinity of chromosomal DSBs, forming microscopically visible, subnuclear foci [19]. These foci assemble to create a “repair-prone” environment [20]. Thus, we assessed whether Netropsin affects the formation of 53BP1 foci during BoHV-1 replication using confocal microscopy. We observed that 53BP1 nuclear foci were rarely present in BoHV-1-infected cells treated with the DMSO control (Figure 4, upper panel), a finding consistent with our previous report (submitted to Veterinary Microbiology, under review). In contrast, typical DNA foci were distinctly observed in virus-infected cells treated with Netropsin, with the zoomed-in images within green frames particularly highlighting the characteristic 53BP1 foci (Figure 4A). Approximately 17.35% of cells treated with 10 μM Netropsin exhibited ≥ 5 53BP1 foci in the context of virus infection (Figure 4A,B). This suggests that virus infection-induced depletion of 53BP1 foci was reversed by Netropsin treatment. Of note, Netropsin at a concentration of 50 μM significantly reduces BoHV-1 productive infection [15], whereas at a concentration of 10 μM it could not obviously affect viral replication, as evidenced by the unchanged expression of virion-associated proteins (Figure 2C). Consequently, the restored formation of 53BP1 foci by Netropsin treatment is not attributable to changes in viral titers.

### 2.5. Netropsin Altered the Accumulation of 53BP1 in the Nucleus, an Effect That Was Reversed by Virus Infection

**Figure 5:**
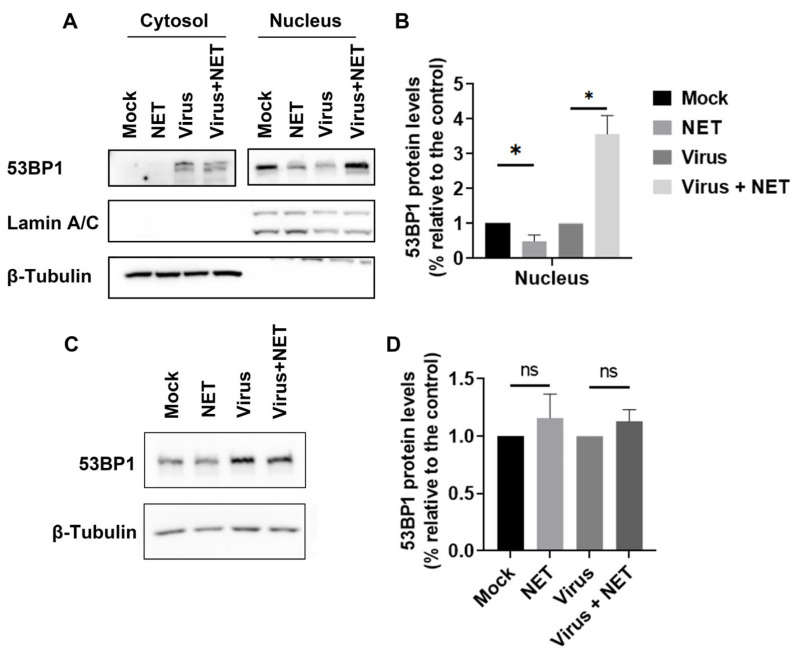
Impact of Netropsin on nuclear accumulation of HMGA1 in MDBK cells. (**A**) MDBK cells in 100 mm dishes were either mock-infected or infected with BoHV-1 at an MOI of 1 for 24 h in the presence of either DMSO control or Netropsin (10 μM). Then, cellular fractions of nucleus and cytosol were purified using a commercial kit (Beyotime Biotechnology, cat# P0027). Protein levels of 53BP1 in both fractions were detected by Western blotting. (**B**,**D**) The band intensity of 53BP1 was analyzed using the free software Image J. The band intensity of 53BP1 in both mock-infected control and virus-infected cells was set to 1 for normalization purposes. The data presented are the means from three independent experiments, with error bars indicating standard deviations. Significance was assessed using Student’s *t*-test (* *p* < 0.05, ns, not significant). (**C**) MDBK cells in 60 mm dishes were either mock-infected or infected with BoHV-1 at an MOI of 1 for 24 h in the presence of either DMSO control or Netropsin (10 μM). Then, cell lysates were prepared and subjected to detection of 53BP1 by Western blotting. Data shown are representative of three independent experiments.

To understand how the formation of 53BP1 foci in the nucleus was significantly reversed by Neropsin treatment, we examined the expression of 53BP1 protein in both cytosol and nucleus fractions using Western blotting. As shown in Figure 5, treatment with Netropsin led to a decrease in the accumulation of 53BP1 protein in nuclear fractions, which was reduced to approximately 48.97% compared to the mock-treated control. However, this reduction was entirely reversed upon virus infection. Quantitative analysis revealed that Netropsin treatment increased the accumulation of 53BP1 protein in the nucleus approximately 3.57-fold relative to the mock-treated control. The successful separation of cytosolic and nuclear fractions was confirmed by the distinct presence of Lamin A/C in the nuclear fractions and β-tubulin in the cytosolic fractions, which serve as specific markers for nuclear and cytosolic proteins, respectively (Figure 5A). We observed that Netropsin treatment did not affect the total protein expression levels of 53BP1, regardless of whether the cells were infected with the virus or not (Figure 5C,D). In addition, at a concentration of 10 μM, Netropsin exhibited no significant effects on the expression of virion-associated proteins, as detected by Western blotting (Figure 5). Collectively, these findings indicate that Netropsin treatment influences the nuclear accumulation of 53BP1 protein, and this effect is not due to changes in the overall expression levels of 53BP1 protein.

### 2.6. siRNA-Mediated Knockdown of HMGA1 Decreases 53BP1 Protein Expression

**Figure 6:**
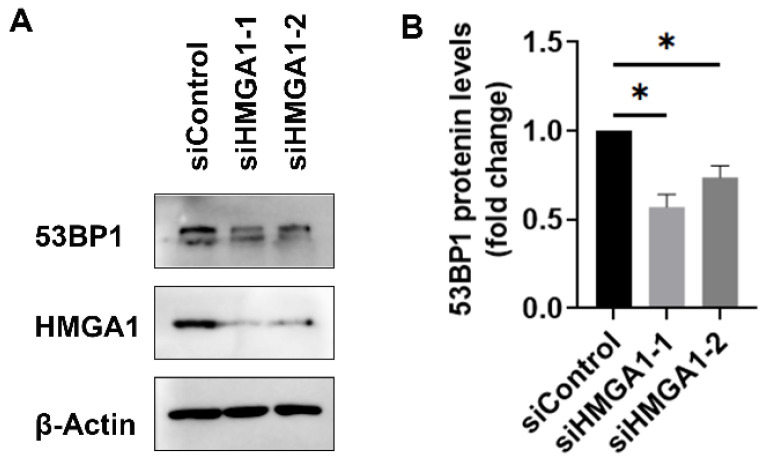
Impact of knockdown HMGA1 on 53BP1 protein expression in MDBK cells. (**A**) MDBK cells in 60 mm dishes were transfected with either scramble siRNA (siControl) or HMGA1-specific siRNAs (siHMGA1-1 and siHMGA1-2). After 48 h of transfection, cell lysates were prepared for the detection of 53BP1 by Western blot. β-Actin was used as a protein load control and for normalization. Data shown are representative of three independent experiments. (**B**) The band intensity of 53BP1 was analyzed using the free software Image J. The band intensity of 53BP1 in transfection of scrambled siRNA control was arbitrarily set to 1 for normalization purposes. The data presented are the means from three independent experiments, with error bars indicating standard deviations. Significance was assessed using Student’s *t*-test (* *p* < 0.05).

Since Netropsin has off-target effects, HMGA1-specific mRNAs were employed to determine whether HMGA1 acts specifically on 53BP1. When HMGA1 protein expression was knocked down by transfection of either siRNAHMGA1-1 or siRNAHMGA1-2, the protein levels of 53BP1 were consistently reduced in comparison to the case with transfection of siRNAControl (Figure 6A). Quantitative analysis indicated that 53BP1 protein levels were decreased to approximately 57.19% and 73.72% by siRNAHMGA1-1 and siRNAHMGA1-2, respectively, relative to the transfected control (Figure 6B). These data corroborated our findings that the knockdown of HMGA1 expression led to an interruption of 53BP1 signaling, which may consequently lead to an exacerbation of DNA damage.

## 3. Discussion

BoHV-1 productive infection induces DNA damage, which may potentially contribute to virus pathogenicity partially via the promotion of cell apoptosis [16,17]. Both viral proteins and host factors have been shown to contribute to the production of DNA damage. For instance, it has been reported that the viral protein VP8 interacts with and affects the function of various DNA damage response (DDR) proteins, such as Ataxia telangiectasia mutated (ATM), phosphorylated Nijmegen breakage syndrome (NBS1), structural maintenance of chromosomes-1 (SMC1), and DNA damage binding protein-1 (DDB1). These interactions result in DNA damage and subsequently lead to cell apoptosis [17,21]. We have previously demonstrated that BoHV-1 productive infection induces an overproduction of reactive oxygen species (ROS), which in turn promotes DNA damage [16]. Here, we showed that HMGA1, a non-histone chromatin regulator, may play a significant role in counteracting virus infection-induced DNA damage, as the siRNA-mediated knockdown of HMGA1 protein expression or inhibition of HMGA1 activity by the chemical inhibitor Netropsin uniformly exacerbates the DNA damage induced by BoHV-1 productive infection (Figure 2 and Figure 3).

Accumulating studies have suggested that HMGA1 plays a significant role in DNA damage repair through distinct mechanisms [22]. It may partially repair damaged DNA through its ability to interact with DNA directly. For instance, HMGA1 aids in DNA damage repair by enabling the completion of homologous recombination and by promoting chromocenter formation [23,24]. Additionally, HMGA1 contributes to DNA damage repair through its transcriptional activities. For example, HMGA1 positively regulates the expression and activation of the DNA damage response protein ATM [5,25]. In this study, we discovered for the first time that HMGA1 positively regulates 53BP1 protein expression (Figure 6), and Netropsin, a potential inhibitor of HMGA1, decreases the nuclear accumulation of 53BP1 protein (Figure 5). This suggests that HMGA1 may influence the biological function of the 53BP1 protein. However, this effect is entirely reversed upon virus infection, indicating that virus infection disrupts the interaction between HMGA1 and 53BP1.

In summary, for the first time we reported that HMGA1 is potentially involved in countering DNA damage induced by virus infection. HMGA1 does indeed modulate the nuclear accumulation of 53BP1 protein, but this effect is counteracted by virus infection. Therefore, the biological function of HMGA1 in countering virus infection-induced DNA damage may be independent of its regulation of 53BP1 signaling. This is the first report suggesting that HMGA1 may be implicated in virus infection-induced DNA damage, although the precise mechanism remains to be elucidated. Furthermore, we report for the first time that HMGA1 positively regulates 53BP1 expression. These findings will expand our understanding of the biological functions of HMGA1 and the mechanisms underlying DNA damage induced by productive BoHV-1 infection.

## 4. Materials and Methods

### 4.1. Cells and Virus

MDBK cells were obtained from the Chinese Model Culture Preservation Center (Shanghai, China). These cells were cultured in DMEM medium containing 10% fetal bovine serum (FBS) (Thermo Fisher Scientific, Waltham, MA, USA). BoHV-1 strain NJ-16-1 was isolated from cow semen in China. The virus was propagated in MDBK cells in a large amount. Then, the aliquots were stored at −80 °C until use.

### 4.2. Antibodies

The antibodies used in this study are detailed below: Lamin A/C monoclonal antibody (mAb) (Santa Cruz Biotechnology, Dallas, TX, USA, cat# sc-376248), β-Tubulin mAb (Abclonal, Wuhan, China, cat#AC051), 53BP1 pAb (NOVUS, Centennial, CO, USA, cat#NB100-304), β-Actin mAb (ProteinTech Group, Rosemont, IL, USA cat#60008-1-Ig), HRP-labeled goat anti-rabbit IgG (Biodragon, Suzhou, China, cat#BF03008), and Alexa Fluor 488^®^-conjugated goat anti-rabbit IgG (H + L) (Waltham, MA, USA, cat# A-11008).

### 4.3. Western Blotting Analysis

Cell lysates were prepared using RIPA buffer (50 mM Tris-HCl, pH 8, 150 mM NaCl, 1% Triton X-100, 0.5% sodium deoxycholate, 0.1% SDS) containing a cocktail of protease and phosphatase inhibitors (Thermo-Scientific). The individual samples were boiled in Laemmli sample buffer for 5 min, separated on an SDS–polyacrylamide gel, and transferred to PVDF membranes. Targeted proteins were probed using a panel of antibodies as described above.

For the designated studies, band intensity was quantitatively analyzed using the free Image J program (https://imagej.net/ij/download.html, accessed on 1 December 2020), which was accessed on 1 December 2020. The band intensity was initially normalized to β-Actin, β-Tubulin, or Lamin A/C, respectively, and the fold change after treatment was calculated. Protein levels in mock-treated cells were arbitrarily set to 1. Significance was assessed using Student’s *t*-test with GraphPad Prism software (v5.0). *p* values of less than 0.05 (* *p* < 0.05) were considered significant for all calculations.

### 4.4. Immunofluorescence Assay (IFA)

Confluent MDBK cells were either mock-infected or infected with BoHV-1 at an MOI of 1 for 24 h. Following two washes with PBS (pH 7.4), cells were fixed with 4% paraformaldehyde for 10 min at room temperature, permeabilized with 0.25% Triton X-100 in PBS (pH 7.4) for 10 min at room temperature, and blocked with 1% BSA in PBST (PBS with 0.1% Tween 20) for 30 min at room temperature. The appropriate primary antibody was diluted in 1% BSA and incubated at 4 °C for 12 h. After three washes, the cells were incubated with Alexa Fluor 488^®^-conjugated goat anti-rabbit IgG (H + L) in the dark for 1 h. After three further washes with PBS, the nuclei were stained with DAPI (4′,6-diamino-2-phenylindole) and the cells were mounted with an anti-fading mounting medium (Electron Microscopy Sciences, Hatfield, PA, USA, catalog number 50-247-04). Images were captured using a Leica confocal microscope(Leica Mikrosysteme, Wetzlar, Hesse, Germany).

### 4.5. Comet Assay

DNA damage was assessed using the alkaline comet assay (single-cell gel electrophoresis), with modifications to the method as described previously [16]. In brief, MDBK cells cultured in 24-well plates were infected with BoHV-1 at an MOI of 0.1. At 12 or 24 hpi, cells were harvested and embedded in low-melting agarose on slides pre-coated with 1% normal-melting agarose, with an additional top layer of low-melting agarose. Cells were lysed in a cold (4 °C) lysis buffer containing 2.5 M NaCl, 100 mM Na2EDTA, 10 mM Tris, 1% Triton X, and 10% DMSO at pH 10.0 for 1 h. The slides were then subjected to horizontal gel electrophoresis in a cold (4 °C) alkaline electrophoresis buffer composed of 300 mM NaOH and 1 mM Na_2_EDTA at pH 12.5, run at 25 V and 300 mA for 40 min. Following electrophoresis, the slides were rinsed twice with neutralization buffer (0.4 M Trizma base, pH 7.5, at 4 °C) for 10 min and allowed to air-dry. DNA was stained with propidium iodide (PI) at a concentration of 20 μg/mL, and images were subsequently captured using a fluorescence microscope.

Approximately three hundred randomly selected cells from each sample were analyzed using CASP software (University of Wroclaw, Wrocław, Poland). The percentage of DNA in the tail (tailDNA%) was employed as the metric for DNA migration.

## Data Availability

The authors declare that all the data are available upon request.
